# Risk factors for postdischarge mortality following hospitalization for severe acute malnutrition in Zimbabwe and Zambia

**DOI:** 10.1093/ajcn/nqaa346

**Published:** 2021-01-20

**Authors:** Mutsa Bwakura-Dangarembizi, Cherlynn Dumbura, Beatrice Amadi, Deophine Ngosa, Florence D Majo, Kusum J Nathoo, Simutanyi Mwakamui, Kuda Mutasa, Bernard Chasekwa, Robert Ntozini, Paul Kelly, Andrew J Prendergast, the HOPE-SAM study team, Ellen Besa, D Bourke Claire, Pamela Chakara, Leah Chidamba, Theodore Chidawanyika, Tafadzwa Chidhanguro, Kapula Chifunda, Esther Chilala, Lovemore Chingaoma, Miyoba Chipunza, Nivea Chulu, Adlight Dandadzi, Tenzeni Dumba, Washington Dune, Temwaninge Gondwe, Margaret Govha, Karen Gwanzura, H Jean Humphrey, Tomola Kaingu, Chanda Kapoma, Sarudzai Kasaru, Lydia Kazhila, Chipo Kureva, Lucy Macwani, Idah Mapurisa, Patience Mashayanembwa, Faithful Masimba, Stephen Moyo, Eddington Mpofu, Mary Mpundu, Edith Mukusho, Johnson Mushonga, Agatha Muyenga, Sophreen Mwaba, Mpala Mwanza, Benjamin Mwapenya, Gwendolyn Nayame, Sibongile Nkiwane, Penias Nyamwino, Evelyn Nyendwa, Dennis Phiri, Phillipa Rambanepasi, Ruairi Robertson, Sandra Rukobo, Thompson Runodamoto, Virginia Sauramba, Shepherd Seremwe, Pururudzai Simango, Jonathan Sturgeon, R Jonathan Swann, Andreck Tembo, Dalitso Tembo, Blessing Tsenesa, C K Jonathan Wells, Kanekwa Zyambo, Khozya Zyambo

**Affiliations:** Department of Paediatrics and Child Health, University of Zimbabwe College of Health Sciences, Harare, Zimbabwe; Zvitambo Institute for Maternal and Child Health Research, Harare, Zimbabwe; Zvitambo Institute for Maternal and Child Health Research, Harare, Zimbabwe; Tropical Gastroenterology and Nutrition Group, University of Zambia, Lusaka, Zambia; Tropical Gastroenterology and Nutrition Group, University of Zambia, Lusaka, Zambia; Zvitambo Institute for Maternal and Child Health Research, Harare, Zimbabwe; Department of Paediatrics and Child Health, University of Zimbabwe College of Health Sciences, Harare, Zimbabwe; Tropical Gastroenterology and Nutrition Group, University of Zambia, Lusaka, Zambia; Zvitambo Institute for Maternal and Child Health Research, Harare, Zimbabwe; Zvitambo Institute for Maternal and Child Health Research, Harare, Zimbabwe; Zvitambo Institute for Maternal and Child Health Research, Harare, Zimbabwe; Tropical Gastroenterology and Nutrition Group, University of Zambia, Lusaka, Zambia; Blizard Institute, Queen Mary University of London, London, UK; Zvitambo Institute for Maternal and Child Health Research, Harare, Zimbabwe; Blizard Institute, Queen Mary University of London, London, UK

**Keywords:** mortality, postdischarge, severe acute malnutrition, HIV, ART, Africa

## Abstract

**Background:**

Children discharged from hospital following management of complicated severe acute malnutrition (SAM) have a high risk of mortality, especially HIV-positive children. Few studies have examined mortality in the antiretroviral therapy (ART) era.

**Objectives:**

Our objectives were to ascertain 52-wk mortality in children discharged from hospital for management of complicated SAM, and to identify independent predictors of mortality.

**Methods:**

A prospective cohort study was conducted in children enrolled from 3 hospitals in Zambia and Zimbabwe between July 2016 and March 2018. The primary outcome was mortality at 52 wk. Univariable and multivariable Cox regression models were used to identify independent risk factors for death, and to investigate whether HIV modifies these associations.

**Results:**

Of 745 children, median age at enrolment was 17.4 mo (IQR: 12.8, 22.1 mo), 21.7% were HIV-positive, and 64.4% had edema. Seventy children (9.4%; 95% CI: 7.4, 11.7%) died and 26 exited during hospitalization; 649 were followed postdischarge. At discharge, 43.9% had ongoing SAM and only 50.8% of HIV-positive children were receiving ART. Vital status was ascertained for 604 (93.1%), of whom 55 (9.1%; 95% CI: 6.9, 11.7%) died at median 16.6 wk (IQR: 9.4, 21.9 wk). Overall, 20.0% (95% CI: 13.5, 27.9%) and 5.6% (95% CI: 3.8, 7.9%) of HIV-positive and HIV-negative children, respectively, died [adjusted hazard ratio (aHR): 3.83; 95% CI: 2.15, 6.82]. Additional independent risk factors for mortality were ongoing SAM (aHR: 2.28; 95% CI: 1.22, 4.25), cerebral palsy (aHR: 5.60; 95% CI: 2.72, 11.50) and nonedematous SAM (aHR: 2.23; 95% CI: 1.24, 4.01), with no evidence of interaction with HIV status.

**Conclusions:**

HIV-positive children have an almost 4-fold higher mortality than HIV-negative children in the year following hospitalization for complicated SAM. A better understanding of causes of death, an improved continuum of care for HIV and SAM, and targeted interventions to improve convalescence are needed.

## Introduction

Malnutrition underlies almost 50% of deaths in children aged <5 y (1). Severe acute malnutrition (SAM) is characterized by muscle wasting with or without edema and is classified into complicated and uncomplicated forms to guide clinical management. Children with a preserved appetite who are clinically well have uncomplicated SAM and are managed in the community. Those with poor appetite, severe edema, medical complications, or ≥1 integrated management of childhood illness danger signs (inability to drink, vomiting everything, convulsions, lethargic, or unconscious) have complicated SAM and are hospitalized (2). Despite standardized treatment guidelines (3), in-hospital mortality of children with complicated SAM in sub-Saharan Africa remains at 10–40% (4–6).

Children are discharged from hospital after resolution of complications and return of appetite to continue community-based nutritional rehabilitation. Emerging data suggest that there is high ongoing mortality risk following hospital discharge (7) and discharge from treatment programs (4). A recent systematic review reported mortality rates between 0.6% and 10.4%, 6–24 mo after discharge from community-based therapeutic feeding centers following nutritional rehabilitation for uncomplicated SAM (8). Some of these studies had high loss to follow-up, implying that actual mortality could be greater (9, 10). It is likely that children hospitalized for complicated SAM have poorer outcomes than those with uncomplicated SAM, but there were too few studies to differentiate mortality risk in these groups.

The ongoing risk of postdischarge mortality in children recovering from complicated SAM is likely due to incomplete nutritional and immune recovery, leading to an elevated risk of death from infections (11–13). In addition, children frequently return to a home environment characterized by poverty, poor water and sanitation, and inadequate health services, which are all recognized underlying determinants of child undernutrition (14). Another major factor underlying mortality in sub-Saharan Africa is concurrent HIV infection (15, 16). HIV has changed the epidemiology, clinical presentation, and pathophysiology of SAM (5), resulting in a 3-fold higher risk of death compared with HIV-negative children prior to the availability of antiretroviral therapy (ART) (5, 17, 18). It remains unclear whether this risk has changed since the introduction of 2013 WHO recommendations to treat all HIV-positive children regardless of clinical or immunological disease stage (19).

There is therefore an urgent need to ascertain risk factors for mortality after discharge from hospital with a view to providing additional support to those at highest risk and improving long-term outcomes. This study reports 1-y postdischarge mortality in children with complicated SAM and determines the risk factors for mortality. Our hypothesis was that children with HIV and SAM have higher mortality than children with SAM alone, despite availability of ART, and that biological and social risk factors predict postdischarge mortality.

## Methods

### Study design

Health Outcomes, Pathogenesis and Epidemiology of Severe Acute Malnutrition (HOPE-SAM) was an observational cohort study in children hospitalized for complicated SAM at 3 urban tertiary referral hospitals in Zambia (University Teaching Hospital) and Zimbabwe (Harare Central and Parirenyatwa hospitals), between July 2016 and March 2019. These hospitals serve a catchment population of urban, periurban, and rural districts. The study design has previously been reported in detail (20). The main study outcomes were mortality, morbidity, and growth over 1 y of follow-up. The protocol, standard operating procedures, and case report forms are available at https://osf.io/29uaw/.

### Study population

Caregivers were given information about the study, and children were enrolled as soon as possible after hospitalization. Eligible children were aged <60 mo and hospitalized with complicated SAM, which was defined as weight-for-height *z*-score (WHZ) <−3, midupper arm circumference (MUAC) <115 mm, or the presence of bilateral edema in children aged >6 mo; and WHZ <−3 or nutritional edema in those aged <6 mo, based on WHO criteria (2). Children who had malignancy, died prior to study enrolment, or whose caregiver was not willing to learn the child's HIV status were ineligible. Study participants hospitalized more than once during the study period were only enrolled during their first admission. The primary outcome was 1-y postdischarge mortality following the index hospitalization.

### Study procedures and follow-up

Baseline clinical, demographic, household, and caregiver data were collected using a standardized case report form. Hospitals implemented country guidelines for provider-initiated counseling and testing for HIV for the caregiver and child; where HIV status was not confirmed in hospital, testing was undertaken by the study. HIV status for mothers and for children aged >18 mo was determined using a rapid antibody test algorithm, and for children <18 mo using HIV DNA PCR. Children were managed by the ward clinical teams and reviewed daily by a study physician during hospitalization to record data on clinical management and progress. Inpatient management of SAM and HIV, and discharge criteria following nutritional rehabilitation, followed WHO-adapted country guidelines (2, 21). Anthropometry was undertaken at baseline, discharge, and follow-up visits. Weight in infants was recorded to the nearest 10 g using an electronic scale (Seca 384) and in older children to the nearest 100 g using an electronic mother-baby scale (Seca 874); both scales were calibrated weekly. MUAC was measured using WHO/UNICEF color-coded tapes to the nearest 1 mm. Length (<2 y or those unable to stand) or height were measured using a UNICEF measuring board.

Children received ready-to-use therapeutic food (RUTF) after discharge in local clinics or hospital outpatient departments, according to country-specific guidelines on management of SAM. Hospital follow-up was conducted in dedicated study clinics at 2, 4, 12, 24, and 48 wk postdischarge, with window periods created around each visit to maximize follow-up. The original window period for the endline visit (44–52 wk) was subsequently extended to 72 wk to maximize follow-up. Mortality analysis was conducted through 52 wk postdischarge. Data were collected at each visit from caregiver interview and review of hand-held medical records where available. Illnesses identified at follow-up visits were managed by study doctors, with referral to hospital if needed. Follow-up phone calls were made to reschedule missed appointments, and home visits were undertaken for chronic defaulters where possible. Vital status was ascertained by telephone calls for those who had defaulted follow-up. All data were captured on paper case report forms and double-entered onto a custom-developed online database, with resolution of queries by study nurses and physicians.

### Sample size

The observational cohort aimed to enrol ≤800 children with complicated SAM. We estimated that a mortality of 15% and overall loss to follow-up of 15% would provide 560 evaluable children at 1 y, of whom 224 would have HIV-SAM based on an estimated prevalence of 40% in hospitalized children (17). This would provide >80% power at 5% significance to detect absolute differences of 17% in binary outcomes between HIV-positive and HIV-negative children with SAM, and of 0.33 SDs in continuous outcomes.

### Statistical methods

A prespecified analysis plan is available at https://osf.io/29uaw/. Baseline variables were compared between HIV-positive and HIV-negative groups using χ^2^ or Fisher exact tests for categorical variables and *t* tests for continuous variables. Anthropometric *z*-scores were calculated using 2006 WHO growth standards. Univariable and multivariable Cox regression models were used to explore associations between exposure variables and postdischarge mortality. The time at risk was calculated from hospital discharge to 52 wk, or the date last seen, reported alive, or the date of death. Selection of exposure variables was based on biological plausibility and the existing literature (20). All models included (regardless of statistical significance) the following a priori variables: age, sex, country, edema at admission, SAM at discharge, and HIV status. SAM at discharge was used as a composite variable because WHZ and MUAC showed collinearity (**Supplemental Figure 1**). Backwards elimination with exit *P* value = 0.1 was undertaken for other risk factors, to produce a final “main effects” model. All available data were included, and no variable was missing for >2.5% of the participants. We investigated and accounted for nonlinearity of effects of continuous predictors using multivariable fractional polynomials. We investigated interactions between each included risk factor and HIV status to ascertain whether HIV modifies the association between risk factors and mortality. Analyses were conducted using Stata version 14.0 (StataCorp).

### Ethical considerations

Ethical approval was obtained from the University of Zambia Biomedical Research Ethics Committee, and the Medical Research Council of Zimbabwe. The ethics committee of Queen Mary University of London provided an advisory review. Primary caregivers gave written informed consent for each participant.

## Results

### Study enrolment

**FIGURE 1:**
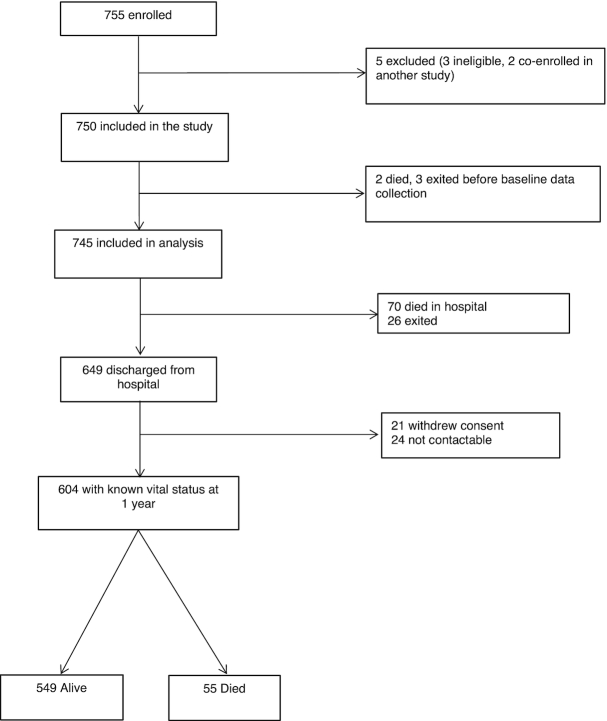
HOPE-SAM (Health Outcomes, Pathogenesis, and Epidemiology of Severe Acute Malnutrition) study flow.

Seven hundred and fifty-five children hospitalized with SAM were enrolled between July 13, 2016 and March 31, 2018. Five children were subsequently excluded from analysis. Among the 750 eligible children, 2 died and 3 withdrew consent prior to baseline data collection (**Figure 1**). Of the 745 children included in the analyses, 37.3%, 32.3%, and 30.3% were from Harare Central Hospital, University Teaching Hospital, and Parirenyatwa Hospital, respectively.

### Baseline characteristics

**Table 1** shows the baseline characteristics of children with SAM, of whom one-fifth were HIV-positive. Of the HIV-negative children, 16.6% were HIV-exposed but uninfected. Just over half of children were male; median age was 17.4 mo (IQR: 12.8, 22.1 mo), with 2.7% aged <6 mo. Almost two-thirds of children had edematous malnutrition. Compared with HIV-negative children, HIV-positive children were significantly older, more wasted, more underweight, more stunted, and less likely to have edema (Table 1). A small proportion of children had chronic underlying conditions. Significantly more HIV-positive compared with HIV-negative children were taking tuberculosis (TB) medication at admission, and just over 40% of HIV-positive children were receiving ART.

**TABLE 1:** Characteristics of children at hospitalization and discharge^1^

	Admission				Discharge			
	All	HIV-positive	HIV-negative	*P* value^2^	All	HIV-positive	HIV-negative	*P* value^2^
	*n* = 745	*n* = 162	*n* = 583		*n* = 649	*n* = 130	*n* = 519	
Male	390/745 (52.4%)	82/162 (50.6%)	308/583 (52.8%)	0.62	344/649 (53.0%)	66/130 (50.8%)	278/519 (53.6%)	0.57
Median age, mo (IQR)	17.4 (12.8, 22.1)	19.3 (13.6, 24.1)	17.1 (12.7, 21.5)	0.002	18.2 (13.6, 22.6)	19.4 (14.3, 24.5)	17.3 (13.2, 21.9)	<0.001
1–5 mo	20/745 (2.7%)	4/162 (2.5%)	16/583 (2.7%)	0.07	17/649 (2.6%)	3/130 (2.3%)	14/519 (2.7%)	0.008
6–11 mo	131/745 (17.6%)	27/162 (16.7%)	104/583 (17.8%)		89/649 (13.7%)	13/130 (10.0%)	76/519 (14.6%)	
12–23 mo	458/745 (61.5%)	90/162 (55.6%)	368/583 (63.2%)		412/649 (63.5%)	74/130 (56.9%)	338/519 (65.2%)	
24–59 mo	136/745 (18.3%)	41/162 (25.3%)	95/583 (16.3%)		131/649 (20.2%)	40/130 (30.8%)	91/519 (17.5%)	
Type of SAM at hospitalization								
Edematous	480/745 (64.4%)	86/162 (53.1%)	394/583 (67.6%)	0.001	53/649 (8.2%)	8/130 (6.2%)	45/519 (8.6%)	0.35
Nonedematous	265/745 (35.6%)	76/162 (46.9%)	189/583 (32.4%)		596/649 (91.8%)	122/130 (93.8%)	474/519 (91.4%)	
Nutritional status at discharge								
SAM	N/A	N/A	N/A	N/A	285/649 (43.9%)	72/130 (55.4%)	213/519 (41.1%)	0.003
No SAM	N/A	N/A	N/A		364/649 (56.1%)	58/130 (44.6%)	306/519 (59.0%)	
Anthropometry								
WHZ, mean ± SD	−2.9 ± 1.8	−3.6 ± 1.8	−2.7 ± 1.8	<0.001	−2.2 ± 1.5	−2.4 ± 1.4	−2.1 ± 1.5	0.05
WAZ, mean ± SD	−3.8 ± 1.8	−4.4 ± 1.5	−3.6 ± 1.8	<0.001	−3.3 ± 1.6	−3.7 ± 1.2	−3.2 ± 1.7	0.002
HAZ, mean ± SD	−3.0 ± 1.6	−3.3 ± 1.3	−2.9 ± 1.6	0.005	−3.1 ± 1.5	−3.5 ± 1.2	−3.0 ± 1.6	0.003
MUAC, mm; mean ± SD	118 ± 17	110 ± 15	120 ± 17	<0.001	123 ± 16	117 ± 14	124 ± 16	<0.001
Chronic underlying conditions								
Cerebral palsy	32/745 (4.3%)	1/162 (0.6%)	31/583 (5.3%)	0.009	30/649 (4.6%)	1/130 (0.8%)	29/519 (5.6%)	0.02
Hydrocephalus	5/745 (0.7%)	0/162 (0.0%)	5/583 (0.9%)	0.24	3/649 (0.5%)	0/130 (0.0%)	3/519 (0.6%)	0.39
Congenital heart disease	14/745 (1.8%)	1/162 (0.6%)	13/583 (2.2%)	0.18	14/649 (2.1%)	1/130 (0.8%)	13/519 (2.5%)	0.22
Cleft palate	4/745 (0.5%)	0/162 (0.0%)	4/583 (0.7%)	0.29	3/649 (0.5%)	0/130 (0.0%)	3/519 (0.6%)	0.39
Medications								
TB medication	24/745 (3.2%)	13/162 (8.0%)	11/583 (1.9%)	<0.001	88/649 (13.6%)	40/130 (30.7%)	48/519 (9.2%)	<0.001
ART medication	N/A	66/162 (40.7%)	N/A	N/A	N/A	66/130 (50.8%)	N/A	N/A
Hemoglobin g/dL; median (IQR)^3^	N/A	N/A	N/A	N/A	9.3 (8.2, 10.3)	8.8 (7.7, 9.6)	9.4 (8.3, 10.4)	<0.001
Anemia^4^								
None (≥11 g/dL)	N/A	N/A	N/A	N/A	97/621 (15.6%)	13/123 (10.5%)	84/498 (16.9%)	0.02
Mild (10–10.9 g/dL)	N/A	N/A	N/A	N/A	102/621 (16.4%)	12/123 (9.8%)	90/498 (18.1%)	
Moderate (7–9.9 g/dL)	N/A	N/A	N/A	N/A	368/621 (59.3%)	84/123 (68.3%)	284/498 (57.0%)	
Severe (<7 g/dL)	N/A	N/A	N/A	N/A	54/621 (8.7%)	14/123 (11.4%)	40/498 (8.0%)	

^1^Data are *n*/total (column %) unless otherwise stated. ART, antiretroviral therapy; HAZ, height-for-age *z*-score; MUAC, midupper arm circumference; N/A: not applicable, SAM, severe acute malnutrition; TB: tuberculosis; WAZ, weight-for-age *z-*score; WHZ, weight-for-height *z-*score.

2 *P* value comparing HIV-positive and HIV-negative groups.

3 Hemoglobin was measured at discharge; if no discharge hemoglobin measurement was available, the value closest to discharge was used.

4 Anemia was defined according to WHO guidelines (2011).

Baseline caregiver and household characteristics are shown in **Supplemental Table 1**. Most children were cared for by their biological mothers. There was no difference in caregiver age by HIV status. Caregivers of HIV-positive compared with HIV-negative children were less likely to be married and had fewer years of formal education. Households of HIV-positive compared with HIV-negative children were more likely to be periurban, with poorer sanitation facilities. There were no other significant differences between groups.

### Discharge characteristics

The median duration of hospitalization was 6 d (IQR: 2, 11 d). Seventy (9.4%; 95% CI: 7.4, 11.7%) children died in hospital, leading to an incident death rate of 5.4 (95% CI: 4.2, 6.8) per 100 child-weeks. Twenty-six children (3.4%) exited during hospitalization, meaning 649 children were followed up postdischarge (Figure 1). At discharge, anthropometry had improved compared with values at hospital admission for WHZ, weight-for-age *z*-score (WAZ), and MUAC; almost half of children had ongoing SAM at the time of discharge, as defined by WHO criteria (Table 1). There was a 4-fold increase in the number of children discharged on TB treatment compared with admission. Only half (50.8%) of the HIV-positive children were discharged on ART; another 48 (36.9%) started ART postdischarge, and 16 (12.3%) never started ART during follow-up.

### Postdischarge mortality

**FIGURE 2:**
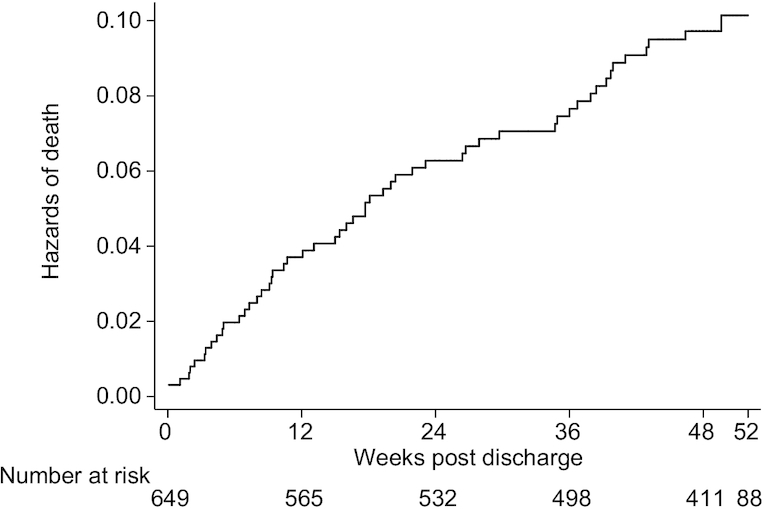
Hazard of death in children discharged after management of complicated severe acute malnutrition. Six hundred and forty-nine children were discharged from hospital, and 448 children attended their endline visit. The target date was 48 wk postdischarge, with an allowable visit window of 44–52 wk, which was subsequently extended to 72 wk to maximize follow-up. For those who did not attend their study visit, a phone call was made during the visit window to ascertain vital status from the primary caregiver. Children were censored as alive at their last contact. If they were seen after 52 wk (*n* = 88; 13.6%), they were censored for analysis at 52 wk. No child died after 52 wk. Twenty-four (3.7%) children were not contactable and were censored when last seen.

Of the 649 children discharged from hospital, vital status was ascertained for 93.1% at 1 y (Figure 1). Baseline characteristics of children with and without available vital status were similar (**Supplemental Table 2**). The median follow-up time was 48.4 wk (IQR: 39.9, 50.9 wk). Of the 55 children known to have died, 32 (58.2%; 95% CI: 44.1, 71.3%) died in the community and 23 (41.8%; 95% CI: 28.7, 55.9%) during a subsequent hospital readmission. The median time to death was 16.6 wk (IQR: 9.4, 21.9) but there was evidence of high ongoing mortality risk throughout follow-up (**Figure 2**). The incident death rate decreased from 0.4 (95% CI: 0.2, 1.0) per 100 child-weeks during weeks 0–2 postdischarge, to 0.15 (95% CI: 0.1, 0.2) per 100 child-weeks during weeks 24–48 postdischarge.

### Risk factors for mortality

**FIGURE 3:**
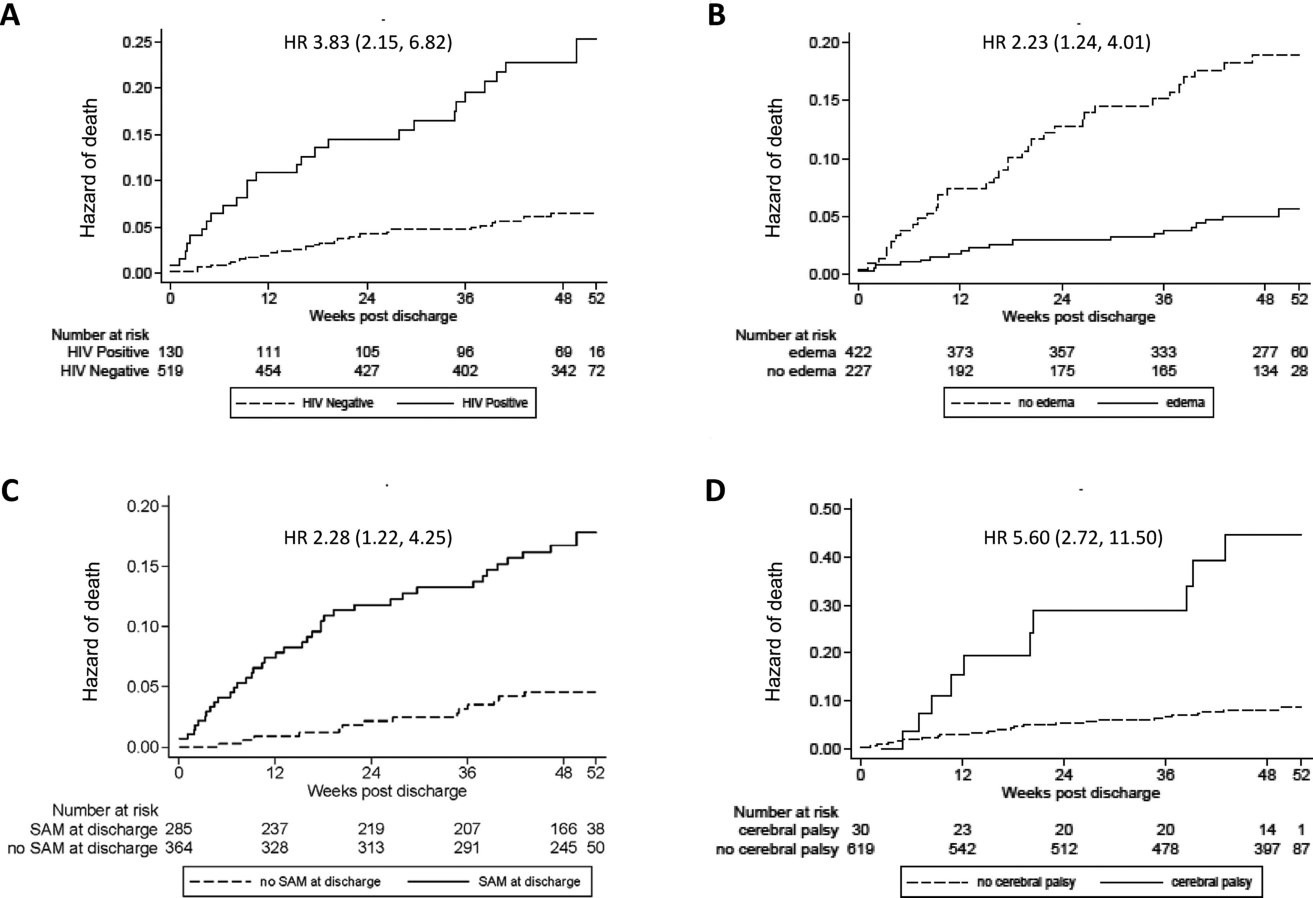
Risk factors for postdischarge mortality. Kaplan–Meier plots showing the hazard of postdischarge death associated with (A) HIV status, (B) edema at hospitalization, (C) ongoing severe acute malnutrition (SAM) at discharge, and (D) cerebral palsy. Adjusted HRs were derived from a multivariable Cox regression model.

Baseline characteristics of children who were alive at the end of follow-up and those who died are shown in **Supplemental Table 3**. Children who died were more wasted (mean WHZ = −3.4 compared with −2.1, *P* < 0.001; mean MUAC = 110 mm compared with 124 mm, *P* < 0.001), underweight (mean WAZ = −4.7 compared with −3.2, *P* < 0.001), and stunted [mean height-for-age *z*-score (HAZ) = −3.7 compared with −3.0, *P* = 0.002], and had been hospitalized longer than those who survived [median 16 d (IQR: 8, 21 d) compared with 12 d (IQR: 7, 14 d), *P* = 0.002]. In the final multivariable model, 4 factors were significant independent predictors of death: HIV, nonedematous malnutrition, SAM at discharge, and cerebral palsy (**Table 2**). HIV-positive compared with HIV-negative children had almost 4-fold higher mortality, with 20.0% (95% CI: 13.5, 27.9%) of HIV-positive children dying compared with 5.6% (95% CI: 3.8, 7.9%) of HIV-negative children [adjusted hazard ratio (aHR): 3.83; 95% CI: 2.15, 6.82] (**Figure 3**A). In HIV-positive children, mortality was not significantly different according to ART status at the time of discharge (HR: 1.09; 95% CI: 0.49, 2.45) (**Supplemental Figure 2**). Children with nonedematous compared with edematous SAM at admission to hospital had twice the mortality risk over the first year postdischarge (aHR: 2.23; 95% CI: 1.24, 4.01) (Figure 3B). Compared with children who did not still have SAM at the time of discharge, those with SAM had >2-fold higher postdischarge mortality (aHR: 2.28; 95% CI: 1.22, 4.25) (Figure 3C). Children with cerebral palsy had an almost 6-fold higher mortality than children without cerebral palsy (aHR: 5.60; 95% CI: 2.72, 11.50) (Figure 3D). Sex, age, duration of hospitalization, or primary caregiver and household characteristics were not associated with postdischarge mortality. There was no evidence of interaction between HIV status and SAM at discharge (*P* = 0.33), cerebral palsy (*P* = 0.22), or edema (*P* = 0.19) in the analyses of postdischarge mortality.

**TABLE 2:** Univariable and multivariable analyses of factors associated with postdischarge mortality^1^

	Survived *n* = 594	Died*n* = 55	Unadjusted HR^2^ (95% CI)	Adjusted HR^3^ (95% CI)	*P* value^4^
Child characteristics					
Country					
Zimbabwe	420 (91.1%)	41 (8.9%)	Reference		
Zambia	174 (92.6%)	14 (7.4%)	0.94 (0.51, 1.72)	0.72 (0.39, 1.35)	0.31
Sex					
Female	275 (90.2%)	30 (9.8%)	Reference		
Male	319 (92.8%)	25 (7.2%)	1.37 (0.81, 2.33)	1.15 (0.67, 1.97)	0.62
Median age at discharge, mo (IQR)	18.4 (13.6, 22.7)	17.0 (10.1, 22.0)	0.97 (0.94, 1.01)	0.98 (0.94, 1.01)	0.17
HIV status					
HIV-negative	490 (94.4%)	29 (5.6%)	Reference		
HIV-positive	104 (80.0%)	26 (20.0%)	3.69 (2.17, 6.26)	3.83 (2.15, 6.82)	<0.001
Type of SAM at hospitalization					
Edematous	402 (95.3%)	20 (4.7%)	Reference		
Nonedematous	192 (84.6%)	35 (15.4%)	3.45 (1.99, 5.97)	2.23 (1.24, 4.01)	0.007
Nutritional status at discharge					
No SAM	349 (95.9%)	15 (4.1%)	Reference		
SAM	245 (85.9%)	40 (14.1%)	3.69 (2.04, 6.68)	2.28 (1.22, 4.25)	0.010
Chronic underlying conditions^5^					
No cerebral palsy	574/619 (92.7%)	45/619 (7.3%)	Reference		
Cerebral palsy^6^	20/30 (66.7%)	10/30 (33.3%)	5.12 (2.58, 10.16)	5.60 (2.72, 11.50)	<0.001

^1^Data are *n*/total (row %) unless otherwise stated. HAZ, height-for-age *z*-score; mfp, multiple fractional polynomial; MUAC, midupper arm circumference; SAM, severe acute malnutrition; TB, tuberculosis; WAZ, weight-for-age *z*-score; WHZ, weight-for-height *z*-score.

2 Unadjusted HRs were estimated using Cox regression analysis.

3 Adjusted HRs were derived from a multivariable fractional polynomial model that contained all other variables in the table.

4 *P* value from mfp Cox regression model.

5 Children who had heart disease and cleft palate excluded due to small numbers.

6 One child had both cerebral palsy and hydrocephalus.

The following variables were offered up to the backward stepwise regression model: site, country, residence, anemia, hemoglobin, sex, age at discharge, HIV status, WHZ at discharge, HAZ at discharge, WAZ at discharge, mean MUAC at discharge, edema at admission, edema at discharge, SAM at discharge, TB at discharge, cerebral palsy, duration of illness, duration of hospitalization, discharge against medical advice, primary caregiver, maternal age, maternal education, caregiver marital status, caregiver employment, water source, type of toilet, electricity, cooking location. The following variables were retained (*P* < 0.1) by the backward stepwise regression procedure: site, country, residence, age at discharge, edema at admission, HAZ at discharge, WAZ at discharge, hemoglobin, HIV status, cerebral palsy, primary caregiver, caregiver marital status, caregiver employment, cooking facilities. These variables were all offered to the final mfp model, except HAZ and WAZ at discharge, which were collinear with SAM at discharge. The table shows the variables that were either retained by the final mfp model at *P* < 0.05, or were forced into the model as a priori variables (age, sex, country, edema at admission, SAM at discharge, and HIV status).

## Discussion

Children hospitalized for complicated SAM have high inpatient mortality, but few studies have reported the ongoing risk of death after hospital discharge. In this longitudinal study from a high HIV prevalence setting, we show that almost 1 in 10 children died in the year following discharge. Mortality was particularly high in HIV-positive children, who had an almost 4-fold higher risk of death than HIV-negative children, regardless of ART initiation. Although the incident death rate fell over time following discharge from hospital, the cumulative mortality was almost 10%, which was similar to inpatient mortality during the index admission. We identified 3 additional independent predictors of mortality: ongoing SAM at the time of discharge, nonedematous malnutrition, and underlying cerebral palsy. The high mortality shown here highlights the long period of vulnerability in children treated for SAM in hospital, particularly in those with HIV infection. Collectively, our data highlight the urgent need for additional interventions during convalescence to improve long-term outcomes of children with complicated SAM.

We specifically set out to stratify postdischarge mortality by HIV status, because studies conducted in the pre-ART era, or when ART initiation was guided by the degree of immunosuppression, reported higher mortality in HIV-positive compared with HIV-negative children with SAM (4, 22, 23). Our findings confirm that postdischarge mortality remains almost 4-fold higher in HIV-positive children, despite guidelines recommending ART when a diagnosis of HIV is made. Malnutrition and HIV interact in a vicious cycle driven by shared biological, immunological, and socioeconomic factors (24, 25). Malnutrition remains one of the commonest presentations of pediatric HIV infection (15, 26–28), with wasting being a risk factor for mortality even after initiation of ART (29). Chronic inflammation, particularly in children with concurrent HIV and SAM, likely contributes to wasting and has been associated with mortality and hospitalization (29, 30). HIV-positive children have greater nutritional needs than HIV-negative children. WHO recommends that calories should be increased by 10% for an asymptomatic HIV-positive child, by 20–30% with chronic illness, and by 50–100% with severe malnutrition, until weight is recovered (31). There are currently no guidelines regarding adjustment to therapeutic feeds for HIV-positive children to accommodate additional energy requirements, and this was not practiced in our study hospitals. There is uncertainty regarding the optimal timing of ART initiation in children with complicated SAM. Clinicians are often concerned about starting ART in sick children due to potential toxicity, immune reconstitution inflammatory syndrome, and poor adherence; however, early ART initiation is safe and feasible (32), and WHO recommends starting ART as soon as possible after stabilization (2). Despite these recommendations, it is striking that only half of HIV-positive children were on treatment at the time of discharge; in those not on ART, one-quarter never started during the subsequent year of follow-up. Mortality did not differ significantly between children who had started or not started ART at discharge. We therefore cannot determine whether inpatient ART initiation would have reduced mortality in this high-risk group, but clearly unnecessary delays arise from onward referral to HIV clinics for ART initiation. Maintaining a continuum of care from hospital discharge into a community-based model offering integrated HIV and nutrition care could improve the outcome of children with these highly interdependent conditions.

Mortality was >2-fold higher in children with SAM at the time of hospital discharge, compared with those who no longer met WHO criteria for SAM. Although most children had lost their edema at the time of discharge, 43.9% had SAM at discharge. Current WHO discharge recommendations from inpatient care are based on resolution of medical and metabolic complications with a view to continuing nutritional rehabilitation as outpatients (2). Data from this cohort confirm the ongoing mortality risk associated with incomplete nutritional recovery. Current treatment protocols do not address underlying causes of malnutrition such as household food insecurity, caregiver wellbeing, and suboptimal home environments. We found no associations between death and caregiver or household characteristics in this cohort, but this could reflect the limited range of variables we evaluated. Factors such as maternal mental health, caregiving capabilities, and access to healthcare were not evaluated. Nearly 60% of deaths in HOPE-SAM occurred in the community, similar to a Ugandan study of postdischarge mortality in children with acute infections (33). This could reflect the impoverished home environment and poor access to functional health systems. Several prior studies have reported that the highest risk of mortality occurs in the first 3 mo postdischarge (4, 34, 35). By contrast, our study showed that the risk of death remained consistent throughout the first year postdischarge. This suggests that if our follow-up period had been >1 y, mortality could have been higher, suggesting a much more protracted window of vulnerability than is generally recognized.

Consistent with several other studies (36, 16, 37–39), HIV-positive compared with HIV-negative children were less likely to have edematous SAM, suggesting that a degree of immune competence is required to generate edema. Nutritional edema is an acute condition, and children with moderate underlying deficits in weight and height but relatively well-preserved body fat appear to respond well to treatment in the absence of complications (40). Wasting, by contrast, is a more long-term process, and children without edema in our cohort had lower anthropometric *z*-scores. The underlying tissue loss and metabolic dysregulation could require a longer period of recovery, leading to an extended window of vulnerability. Cerebral palsy affected a minority of children but was associated with an almost 6-fold higher mortality; one-third of children with cerebral palsy died after discharge from hospital. Many children with cerebral palsy have inherent feeding difficulties and could require more targeted support following hospital discharge.

This is one of the largest longitudinal studies of children hospitalized for complicated SAM to date. We achieved a high ascertainment of vital status 1 y after discharge through cellphone contact and home visits in an extremely mobile population. We prespecified that we would stratify outcomes by HIV status, whereas many studies have not specifically ascertained HIV status in children with SAM (4, 35). Our study has limitations: in particular, we did not have data on several potential risk factors for mortality, including birth weight, gestational age, health-seeking behavior, adherence to RUTF, and coinfections. Where hemoglobin was not measured at the time of discharge, we used values measured closer to the time of admission. Severe anemia during hospitalization has been identified as a risk factor for postdischarge mortality in sub-Saharan Africa regardless of underlying malnutrition (41, 42); whereas most children in this cohort were anemic, <10% had severe anemia, which could have influenced our results. Lastly, our hospital study did not collect data on community-based nutritional rehabilitation postdischarge, which could have influenced mortality.

In summary, postdischarge mortality over 1 y following management of complicated SAM remains unacceptably high, and is similar to inpatient mortality. Overall, 1 in 5 children managed for complicated SAM died either in hospital or in the community. Mortality was almost 4-fold higher in HIV-positive compared with HIV-negative children. Thus, efforts to eliminate mother-to-child HIV transmission and identify HIV-positive infants early are critical to improving health outcomes. Furthermore, ART-naïve children with HIV and complicated SAM should start ART during hospitalization to avoid missing a window of opportunity, and be discharged to a clearly defined and functional continuum of integrated care. ART alone appears insufficient to reduce the excess postdischarge mortality in HIV-positive children with SAM, and additional intervention strategies should be evaluated. Almost half the children in this study were discharged from hospital with ongoing SAM, and had twice the mortality risk compared with children who no longer met the criteria for SAM following inpatient nutritional rehabilitation. Ensuring that the necessary resources and services for outpatient care are available and accessible is important to bridge this period of vulnerability. We found that children in our setting with nonedematous SAM and with underlying cerebral palsy are particularly vulnerable and require more intensive follow-up. Further study into the causes of postdischarge death, and the caregiver and home environment, would inform a more comprehensive package of care to reduce the unacceptably high burden of postdischarge mortality in children with complicated SAM.

## Supplementary Material

nqaa346_Supplemental_File

## Data Availability

Data described in the manuscript, code book, and analytic code will be made available upon request.

## Notes

Members of the HOPE-SAM study team are listed in the Acknowledgments section.

Supported by Medical Research Council (grant MR/K012711/1), Wellcome (grants 107634/Z/15/Z and 108065/Z/15/Z), and a joint award from Wellcome and the Royal Society (206225/Z/17/Z). The funding bodies had no role in the study design, implementation, or analysis and interpretation of the data.

Supplemental Tables 1–3 and Supplemental Figures 1 and 2 are available from the “Supplementary data” link in the online posting of the article and from the same link in the online table of contents at https://academic.oup.com/ajcn/.

Data described in the manuscript, code book, and analytic code will be made available upon request.

Abbreviations used: aHR, adjusted hazard ratio; ART, antiretroviral therapy; HOPE-SAM, Health Outcomes, Pathogenesis, and Epidemiology of Severe Acute Malnutrition; MUAC, midupper arm circumference; RUTF, ready-to-use therapeutic food; SAM, severe acute malnutrition; TB, tuberculosis; WAZ, weight-for-age *z*-score; WHZ, weight-for-height *z*-score.
