# Supplementary material for: Risk factors for postdischarge mortality following hospitalization for severe acute malnutrition in Zimbabwe and Zambia
Source: Am J Clin Nutr. 2021 Jan 20;113(3):665–74. doi: 10.1093/ajcn/nqaa346 (PMC7948837; doi:10.1093/ajcn/nqaa346)

**On-line Supplementary material**

**Title: Risk factors for post-discharge mortality following hospitalization for severe acute malnutrition in Zimbabwe and Zambia**

Mutsa Bwakura-Dangarembizi.

**Supplementary Table 1 Baseline Caregiver and Household Characteristics**

|  | **All**  **N=745^1^** | **HIV-positive**  **N=162** | **HIV-negative**  **N=583** | **P-value^2^** |
| --- | --- | --- | --- | --- |
| **Primary caregiver**   - Mother | 684/727 (94.1%) | 147/156 (94.2%) | 537/571 (94.0%) | 0.90 |
| - Other | 43/727 (5.9%) | 9/156 (5.8%) | 34/571 (5.9%) |  |
| **Age, years;** |  |  |  |  |
| **median (IQR**) | 26 (22, 31) | 28 (23, 32) | 26 (22, 30) | 0.07 |
| 15-19 years | 67/722 (9.3%) | 9/156 (5.8%) | 58/566 (10.2%) | 0.11 |
| 20-39 years | 625/722 (86.6%) | 143/156 (91.7%) | 482/566 (85.2%) |  |
| ≥40 years | 30/722 (4.2%) | 4/156 (2.5%) | 26/566 (4.6%) |  |
| **Marital status** |  |  |  |  |
| - Married or stable union | 571/727 (78.5%) | 104/156 (66.7%) | 430/571 (75.3%) | 0.03 |
| - Other | 156/727 (21.5%) | 52/156 (33.3%) | 141/571 (24.6%) |  |
| **Education, years;**  **median (IQR)** | 10 (7, 11) | 9 (7, 11) | 10 (8, 11) | 0.03 |
| **Employment** |  |  |  |  |
| - None | 431/723 (59.6%) | 84/156 (53.8%) | 347/567 (61.2%) | 0.08 |
| - skilled | 53/723 (7.3%) | 9/156 (5.7%) | 44/567 (7.8%) |  |
| - unskilled | 239/723 (33.1%) | 63/156 (40.4%) | 176/567 (31.0%) |  |
| **Residence** |  |  |  |  |
| - Rural | 113/739 (15.3%) | 25/161 (15.5%) | 88/578 (15.2%) | 0.02 |
| - Urban | 451/739 (61.0%) | 85/161 (52.7%) | 366/578 (63.3%) |  |
| - Peri-urban | 175/739 (23.7%) | 51/161 (31.8%) | 124/578 (21.5%) |  |
| **Drinking water source**   - Improved | 689/736 (93.6%) | 153/161 (95.0%) | 536/575 (93.2%) | 0.41 |
| - Unimproved | 47/736 (6.4%) | 8/161 (5.0%) | 39/575 (6.8%) |  |
| **Toilet facilities**   - Improved | 651/738 (88.2%) | 130/160 (81.2%) | 521/578 (90.1%) | 0.004 |
| - Unimproved | 57/738 (7.7%) | 22/160 (13.8%) | 35/578 (6.1%) |  |
| - None | 30/738 (4.1%) | 8/160 (5.0%) | 22/578 (3.8%) |  |
| **Household electricity**  Yes | 344/728 (47.3%) | 73/157 (46.4%) | 271/571 (47.4%) | 0.83 |
| **Cooking location** |  |  |  |  |
| - Indoors | 431/727 (59.3%) | 90/158 (56.9%) | 341/569 (59.9%) | 0.80 |
| - Outdoors | 145/727 (19.9%) | 33/158 (20.9%) | 112/569 (19.7%) |  |
| - Both | 151/727 (20.8%) | 35/158 (22.2%) | 116/569 (20.4%) |  |

Data are n/N (column %) unless stated.

^1^5 children did not have baseline data collected because they died or their caregiver withdrew consent between enrolment and baseline data collection.

^2^P value comparing HIV-positive and HIV-negative groups.

IQR: Interquartile range; SD: standard deviation; SAM: severe acute malnutrition

**Supplementary Table 2: Baseline characteristics of children with and without data on one-year vital status**

|  | **Vital status known**  **N=604** | **Vital status unknown**  **N=45** | **P value^1^** |
| --- | --- | --- | --- |
| **Child Characteristics** | | |  |
| **Gender**   - Male | 324/604 (53.6%) | 20/45 (44.4%) | 0.23 |
| - Female | 280/604 (46.4%) | 25/45 (55.6%) |  |
| **Age at discharge,** months |  |  |  |
| ; median (IQR) | 18.2 (13.6, 22.5) | 17.8 (14.2, 26.3) | 0.11 |
| - 1-5 months | 17/604 (2.8%) | 0/45 (0.0%) | 0.20 |
| - 6-11 months | 83/604 (13.7%) | 6/45 (13.3%) |  |
| - 12-23 months | 387/604 (64.1%) | 25/45 (55.6%) |  |
| - 24-59 months | 117/604 (19.4%) | 14/45 (31.1%) |  |
| **Hemoglobin,** g/dL; median (IQR)^2^ | 9.3 (8.1, 10.3) | 9.4 (8.4, 10.2) | 0.64 |
| **Anaemia^3^:**   - None | 90/578 (15.6%) | 7/43 (16.3%) | 0.81 |
| - Mild | 95/578 (16.4%) | 7/43 (16.3%) |  |
| - Moderate | 341/578 (59.0%) | 27/43 (62.8%) |  |
| - Severe | 52/578 (9.0%) | 2/43 (4.7%) |  |
| **Nutritional status at discharge**   - SAM | 266/604 (44.0%) | 19/45 (42.2%) | 0.81 |
| - No SAM | 338/604 (56.0%) | 26/45 (57.8%) |  |
| **Anthropometry at discharge**  WHZ, mean (SD) | -2.2 (1.5) | -2.1 (1.5) | 0.53 |
| Not wasted | 276/604 (45.7%) | 23/45 (51.2%) | 0.78 |
| Wasted (WHZ<-2) | 165/604 (27.3%) | 11/45 (24.4%) |  |
| Severely wasted (<-3) | 163/604 (27.0%) | 11/45 (24.4%) |  |
| WAZ, mean (SD) | -3.3 (1.6) | -3.2 (1.7) | 0.62 |
| Not underweight | 106/604 (17.5%) | 8/45 (17.7%) | 0.88 |
| Underweight (WAZ -2 to -3) | 155/604 (25.7%) | 13/45 (28.9%) |  |
| Severely underweight (WAZ <-3) | 343/604 (56.8%) | 24/45 (53.3%) |  |
| HAZ, mean (SD) | -3.1 (1.5) | -3.1 (1.6) | 0.87 |
| Not stunted | 134/604 (22.2%) | 11/45 (24.4%) | 0.77 |
| Stunted (HAZ<-2) | 168/604 (27.8%) | 14/45 (31.2%) |  |
| Severely stunted (HAZ<-3) | 302/604 (50.0%) | 20/45 (44.4%) |  |
| MUAC, mm; mean (SD) | 123 (16) | 125 (15) | 0.23 |
| <115mm | 184/604 (30.5%) | 12/45 (26.7% ) | 0.82 |
| 115 to <125mm | 258/604 (42.7%) | 21/45 (46.6%) |  |
| >125mm | 162/604 (26.8%) | 12/45 (26.7%) |  |
| **Duration of hospitalization**, days; median (IQR) | 7 (4, 12) | 6 (3, 9) | 0.09 |
| **HIV status**   - Positive | 128/604 (21.2%) | 2/45 (4.4%) | 0.007 |
| - Negative | 476/604 (78.8%) | 43/45 (95.6%) |  |
| **TB medication at discharge**   - Yes | 84/604 (13.9%) | 4/45 (8.9%) | 0.34 |
| - No | 520/604 (86.1%) | 41/45 (91.1%) |  |
| **Primary caregiver characteristics** | | |  |
| **Primary caregiver**   - Mother | 547/590 (92.7%) | 42/43 (97.6%) | 0.22 |
| - Other | 43/590 (7.3%) | 1/43 (2.3%) |  |
| **Age**, years; median (IQR) | 26 (22, 31) | 25 (21, 29) | 0.16 |
| **Marital status**   - Married/stable union | 441/589 (74.9%) | 30/44 (68.2%) | 0.33 |
| - Other | 148/589 (25.1%) | 14/44 (31.8%) |  |
| **Education**, years; median (IQR) | 10 (8, 11) | 10 (7, 11) | 0.84 |
| **Employment**   - None | 346/586 (59.0%) | 27/44 (61.4%) | 0.60 |
| - Skilled | 45/586 (7.7%) | 1/44 (2.3%) |  |
| - Unskilled | 195/586 (33.3%) | 16/44 (36.3%) |  |
| **Household Characteristics** | | |  |
| **Residence**   - Rural | 94/602 (15.6%) | 5/43 (11.6%) | 0.56 |
| - Urban | 378/602 (62.8%) | 26/43 (60.5%) |  |
| - Peri-urban | 130/602 (21.6%) | 12/43 (27.9%) |  |
| **Drinking water source**   - Improved | 559/597 (93.6%) | 40/44 (90.9%) | 0.48 |
| - Unimproved | 38/597 (6.4%) | 4/44 (9.1%) |  |
| **Toilet facilities**   - Improved | 533/600 (88.8%) | 35/43 (81.4%) | 0.31 |
| - Unimproved | 46/600 (7.7%) | 5/43 (11.6%) |  |
| - None | 21/600 (3.5%) | 3/43 (7.0%) |  |
| **Electricity in the home**   - Yes | 310/590 (52.5%) | 29/44 (65.9%) | 0.09 |
| - No | 280/590 (47.5%) | 15/44 (34.1%) |  |

Data are n/N (column %) unless stated.

^1^P value comparing vital status known and vital status unknown groups.

^2^Hemoglobin was measured at discharge; if no discharge hemoglobin measurement was available, the value closest to discharge was used.

^3^Anemia was defined according to WHO guidelines (2011) as: none > 11g/dL, mild 10-10.9 g/dL, moderate 7-9.9 g/dL, and severe <7 g/dL.

IQR: Interquartile range; HAZ: height-for-age Z-score; MUAC: mid-upper arm circumference; WAZ: weight-for-age Z score; WHZ: weight-for-height Z score; SD: standard deviation; SAM: severe acute malnutrition; TB: tuberculosis

**Supplementary Table 3 Characteristics of children who died and survived post-discharge**

|  | **Survived**  **N=594** | **Died**  **N=55** |
| --- | --- | --- |
| **Child Characteristics** | | |
| **Age at discharge,** months; median (IQR) | 18.4 (13.6, 22.7) | 17.0 (10.1, 22.0) |
| - 1-5 months | 12/17 (70.5%) | 5/17 (29.5%) |
| - 6-11 months | 79/89 (88.8%) | 10/89 (11.2%) |
| - 12-23 months | 382/412 (92.7%) | 30/412 (7.3%) |
| - 24-59 months | 121/131 (92.4%) | 10/131 (7.6%) |
| **Hemoglobin,** g/dL; median (IQR) | 9.3 (8.2, 10.3) | 9.4 (8.1, 10.3) |
| **Anemia** |  |  |
| - None | 90/97 (92.7%) | 7/97 (7.3%) |
| - Mild | 89/102 (87.3%) | 13/102 (12.7%) |
| - Moderate | 344/368 (93.5%) | 24/368 (6.5%) |
| - Severe | 44/54 (81.5%) | 10/54 (18.5%) |
| **Nutritional status at discharge**   - SAM | 245/285 (85.9%) | 40/285 (14.1%) |
| - No SAM | 349/364 (95.9%) | 15/364 (4.1%) |
| **Anthropometry at discharge**  WHZ; mean (SD) | -2.1 (1.4) | -3.4 (1.5) |
| Not wasted (WHZ>-2) | 291/299 (97.3%) | 8/299 (2.7%) |
| Moderately wasted (WHZ -2 to -3) | 163/176 (92.6%) | 13/176 (7.4%) |
| Severely wasted (WHZ <-3) | 140/174 (80.5%) | 34/174 (19.5%) |
| **WAZ**; mean (SD) | -3.2 (1.5) | -4.7 (1.7) |
| Not underweight (WAZ>-2) | 113/114 (99.1%) | 1/114 (0.9%) |
| Underweight (WAZ -2 to -3) | 164/168 (97.6%) | 4/168 (2.4%) |
| Severely underweight (WAZ <-3) | 317/367 (86.4%) | 50/367 (13.6%) |
| **HAZ**; mean (SD) | -3.0 (1.5) | -3.7 (1.6) |
| Not stunted (HAZ >-2) | 139/145 (95.9%) | 6/145 (4.1%) |
| Stunted (HAZ -2 to -3) | 165/182 (90.7%) | 17/82(9.3%) |
| Severely stunted (HAZ<-3) | 290/322 (90.1%) | 32/322 (9.9%) |
| **MUAC**, mm; mean (SD) | 124 (15) | 110 (14) |
| >125mm | 160/174 (92.0%) | 14/174 (8.0%) |
| 115mm to 125mm | 270/279 (96.8%) | 9/279 (3.2%) |
| <115mm | 164/196 (83.7%) | 32/196 (16.3%) |
| **Duration of hospitalization**, days; median (IQR) | 12 (7, 14) | 16 (8, 21) |
| **TB medication at discharge**   - Yes | 74/88 (84.1%) | 14/88 (15.9%) |
| - No | 520/561 (92.7%) | 41/561 (7.3%) |
| **Primary caregiver characteristics** | | |
| **Primary caregiver**   - Mother | 536/590 (90.8%) | 54/590 (9.2%) |
| - Other | 42/43 (97.7%) | 1/43 (2.3%) |
| **Age,** years; median (IQR) | 26 (22, 31) | 28 (23, 34) |
| **Marital status**   - Married/stable union | 430/471 (91.3%) | 41/471 (8.7%) |
| - Other | 148/162 (91.4%) | 14/162 (8.6%) |
| **Education**, years; median (IQR) | 10 (8, 11) | 10 (7, 11) |
| **Employment**   - None | 344/373 (92.2%) | 29/373 (7.8%) |
| - skilled | 38/46 (82.6%) | 8/46 (17.4%) |
| - unskilled | 193/211 (91.5%) | 18/211 (8.5%) |
| **Household Characteristics** | | |
| **Residence**   - Rural | 91/99 (91.9%) | 8/99 (8.1%) |
| - Urban | 368/404 (91.1%) | 36/404 (8.9%) |
| - Peri-urban | 131/142 (92.3%) | 11/142 (7.7%) |
| **Drinking water source**   - Improved | 548/599 (91.5%) | 51/599 (8.5%) |
| - Unimproved | 38/42 (90.5%) | 4/42 (9.5%) |
| **Toilet facilities**   - Improved | 519/568 (91.4%) | 49/568 (8.6%) |
| - Unimproved | 46/51 (90.2%) | 5/51 (9.8%) |
| - None | 23/24 (95.8%) | 1/24 (4.2%) |
| **Electricity in the home**   - Yes | 272/295 (92.2%) | 23/295 (7.8%) |
| - No | 311/339 (91.7%) | 28/339 (8.3%) |

Data are n (row %) unless stated

IQR: Interquartile range; HAZ: height-for-age Z-score; MUAC: mid-upper arm circumference; SAM: severe acute malnutrition; SD: standard deviation; WAZ: weight-for-age Z score; WHZ: weight-for-height Z score;

**Supplementary Table 4 Mortality incidence rates for the post-discharge follow-up period**

| **Follow-up period** | **Incidence rate per 100 child-weeks (95%CI)** |
| --- | --- |
| 0 to 2 weeks | 0.4 (0.2, 1.0) |
| 2 to 4 weeks | 0.3 (0.1, 0.9) |
| 4 to 12 weeks | 0.3 (0.2, 0.5) |
| 12 to 24 weeks | 0.2 (0.1, 0.4) |
| 24 to 52 weeks | 0.15 (0.1, 0.2) |

**Supplementary Figure 1: Correlation between mid-upper arm circumference and weight-for-height Z-score at discharge**


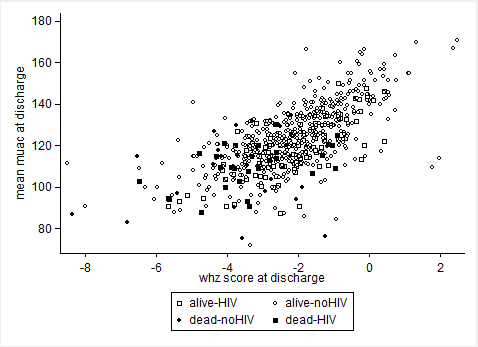


Data are shown for all children who were discharged with available mid-upper arm circumference (MUAC) and weight-for-height Z-score (WHZ) data, split by HIV status and vital status by one year post-discharge.

**Supplementary Figure 2: Hazard of post-discharge mortality among HIV-positive children according to ART status at discharge**


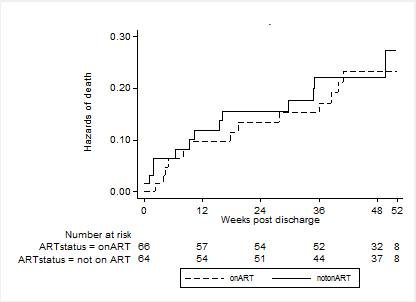

Supplement: nqaa346_Supplemental_File [file nqaa346_supplemental_file.docx]
